# The complete mitochondrial genome of a tropical sea cucumber, *Holothuria leucospilota*

**DOI:** 10.1080/23802359.2019.1668309

**Published:** 2019-09-19

**Authors:** Chuhang Cheng, Zonghe Yu, Chunhua Ren, Xiao Jiang, Xin Zhang, Xiaofen Wu, Wen Huang, Chaoqun Hu

**Affiliations:** a CAS Key Laboratory of Tropical Marine Bio-resources and Ecology (LMB)/Guangdong Provincial Key Laboratory of Applied Marine Biology (LAMB), South China Sea Institute of Oceanology, Chinese Academy of Sciences, Guangzhou China;; b University of Chinese Academy of Sciences, Beijing, China;; c Institution of South China Sea Ecology and Environmental Engineering (ISEE), Chinese Academy of Sciences, Guangzhou, China

**Keywords:** Mitochondrial genome, *Holothuria leucospilota*, phylogenetic tree, PCGs

## Abstract

The mitochondrial genome of *Holothuria leucospilota* was 15,906 bp in length, containing 13 protein-coding genes (PCGs), 22 tRNA genes, and two rRNA genes. There were four initiation codons (ATG, ATT, ATC, and ATA) for the PCGs, and the termination codon of most PCGs was TAA, except for *nad4* (TAG) and *nad6* (TAG). Only one PCG (*nad6*) and five tRNA genes (*tRNA^Ser(UCN)^*, *tRNA^Gln^*, *tRNA^Ala^*, *RNA^Val^*, and *tRNA^Asp^*) were encoded on the light chain; the other genes were encoded on the heavy chain. *H. leucospilota* was most closely related to *Holothuria scabra* in a phylogenetic tree.

*Holothuria leucospilota* (Echinodermata: Holothuroidea, *H. leucospilota*) is a tropical sea cucumber species with a nocturnal habit (Liao 1997). It is widely distributed in the Indo-Western Pacific region (Huang et al. 2018). *H. leucospilota* can accelerate the cycling of calcium carbonate and bioturbation (Benavides-Serrato et al. 2013) to maintain a healthy coral reef ecosystem (Rhoads 1973).

The identification of sea cucumber species based on their mitogenomes would be more accurate than identifying them on the basis of their phenotypic characteristics, such as tentacles, calcareous ring and endoskeleton (Massin 2007; Liao 1997), due to its conserved structure and active primary structure evolution (Boore 1999).

The specimen was collected from Shenzhen, Guangdong province, China (N22°35′, E114°31′), and stored in the Marine Biotechnology and Disease Control Laboratory of the South China Sea at the Chinese Academy of Sciences in Guangzhou, China (MBDC170608102). Total DNA was isolated using the TIANamp Marine Animals DNA Kit (Tiangen Biochemistry Technology Co., Ltd., China), and sequenced using Illumina sequencing (Genomics Co., Ltd., in China). Paired-end reads were aligned by BLAST and spliced using SeqMan software (Fan et al. 2011). BLAST (http://www.ncbi.nlm.nih.gov/BLAST/) and MITOS Web Server BETA (http://bloodymary.bioinf.uni-leipzig.de/mitos/index.py) were used to identify protein-coding genes. tRNA genes were identified by tRNAscan-SE 1.31 software (Lowe and Eddy 1997). A phylogenetic tree was constructed using MEGA 7.0 software.

The mitogenome of *H. leucospilota* (MK801674) showed a closed-ring structure with a total length of 15,906 bp (31.8% A, 25.9% T, 25.8% C, and 16.5% G). The total length of 22 tRNA was 1514 bp, varied from 61 bp (*tRNA^Lys^*) to 72 bp (*tRNA^Leu(UUR)^*) in length. The lrRNA length was 1475 bp, and the srRNA length was 830 bp, accounting for 9.27% and 5.22% of the total length, respectively. 13 PCGs consisted of 3786 codons and accounted for 71.41% of the total length of the genome. The longest noncoding sequence was between *tRNA^Thr^* and *tRNA^Pro^* and acts as the control region, modulating the signals that regulate and initiate mtDNA replication and transcription (Wolstenholme 1992; Shadel and Clayton 1997).

The phylogenetic tree demonstrated that *H. leucospilota* is most closely related to *Holothuria scabra* and that the interspecific relationships among holothuroids are monophyletic (Figure 1). It also implies that they diverged recently from their common ancestor and evolved in a short period of time.

**Figure 1.**
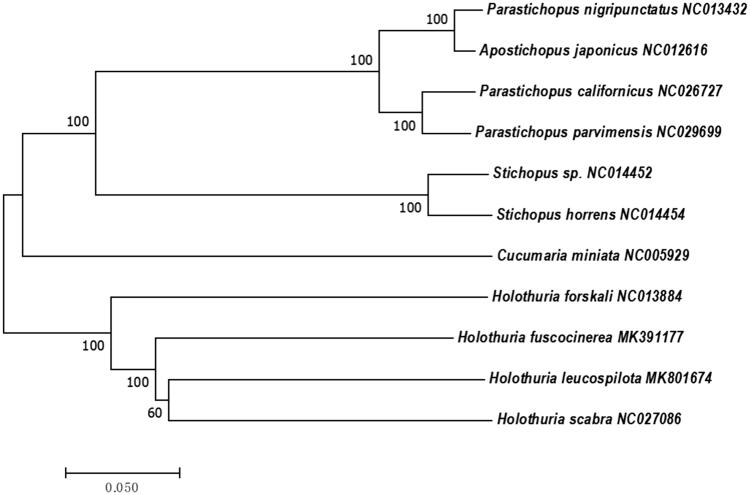
Neighbour-joining phylogenetic tree of *Holothuria leucospilota* and 10 other closely related species based on the full length of mitochondrial genomes.
